# BreastDefend enhances effect of tamoxifen in estrogen receptor-positive human breast cancer in vitro and in vivo

**DOI:** 10.1186/s12906-017-1621-7

**Published:** 2017-02-16

**Authors:** Shujie Cheng, Victor Castillo, Matt Welty, Mark Alvarado, Isaac Eliaz, Constance J. Temm, George E. Sandusky, Daniel Sliva

**Affiliations:** 1 Cancer Research Laboratory, Methodist Research Institute, Indiana University Health, Indianapolis, IN 46202 USA; 2 Amitabha Medical Clinic and Healing Center, Santa Rosa, CA 95401 USA; 3 Department of Pathology, Indiana University School of Medicine, Indianapolis, IN 46202 USA; 4 Department of Medicine, Indiana University School of Medicine, Indianapolis, IN 46202 USA; 5 DSTest Laboratories, Purdue Research Park, 5225 Exploration Drive, Indianapolis, IN 46241 USA; 6 Present address: Department of Food Quality and Safety, School of Engineering, China Pharmaceutical University, Nanjing, People’s Republic of China

**Keywords:** Polybotanical supplement, BreastDefend, Tamoxifen, Estrogen receptor, MCF-7, Xenograft model, Apoptosis

## Abstract

**Background:**

Tamoxifen (TAM) has been widely used for the treatment of estrogen receptor (ER)-positive breast cancer and its combination with other therapies is being actively investigated as a way to increase efficacy and decrease side effects. Here, we evaluate the therapeutic potential of co-treatment with TAM and BreastDefend (BD), a dietary supplement formula, in ER-positive human breast cancer.

**Methods:**

Cell proliferation and apoptosis were determined in ER-positive human breast cancer cells MCF-7 by MTT assay, quantitation of cytoplasmic histone-associated DNA fragments and expression of cleaved PARP, respectively. The molecular mechanism was identified using RNA microarray analysis and western blotting. Tumor tissues from xenograft mouse model were analyzed by immunohistochemistry.

**Results:**

Our data clearly demonstrate that a combination of 4-hydroxytamoxifen (4-OHT) with BD lead to profound inhibition of cell proliferation and induction of apoptosis in MCF-7 cells. This effect is consistent with the regulation of apoptotic and TAM resistant genes at the transcription and translation levels. Importantly, TAM and BD co-treatment significantly enhanced apoptosis, suppressed tumor growth and reduced tumor weight in a xenograft model of human ER-positive breast cancer.

**Conclusion:**

BD sensitized ER-positive human breast cancer cells to 4-OHT/TAM treatment in vitro and in vivo. BreastDefend can be used in an adjuvant therapy to increase the therapeutic effect of tamoxifen in patients with ER-positive breast cancer.

**Electronic supplementary material:**

The online version of this article (doi:10.1186/s12906-017-1621-7) contains supplementary material, which is available to authorized users.

## Background

As the leading cause of cancer death in females, breast cancer is a heterogeneous disease that can be divided into three major subtypes: hormone (estrogen/progesterone) receptor-positive, HER2-positive, and triple-negative (estrogen, progesterone receptor and HER2-negative) [1, 2]. Among them, estrogen receptor (ER)-positive breast tumors comprise approximately 75%, depending on estrogen signaling for growth and survival [3, 4].

Specific subtypes of breast cancer have different responses to therapies and tamoxifen (TAM) is the most commonly used endocrine therapy in treatment of ER-positive breast cancer. TAM is a selective ER modulator and its active metabolite, 4-hydroxytamoxifen (4-OHT), acts as an estrogen antagonist in breast cells that binds ER and blocks its activity to halt cell proliferation and induce apoptosis [5–7]. Unfortunately, *de novo* or acquired resistance occurs in around 30% of all ER-positive breast cancer and tumor recurrence is observed in many patients [8, 9]. Furthermore, long-term administration of TAM may lead to serious side effects, such as fatigue, painful joints and mood changes [10, 11]. Therefore, in order to improve efficacy of the treatment and increase the quality of life, effective adjuvant therapies are urgently required.

Numerous studies support that natural compounds or dietary agents, presented in vegetables, fruits and mushrooms, can affect various molecular targets and signaling pathways leading to their possible use in the combination therapy of breast cancer [6, 12–14]. BreastDefend® (BD) is a dietary supplement formula, which contains extracts from medicinal mushrooms (*Ganoderma lucidum, Coriolus versicolor, Phellinus linteus*), herbs (*Curcuma longa,* S*cutellaria barbata, Astragalus membranaceus*), and purified biologically active components (3, 3′-diindolylmethane, quercetin). These natural agents in BD demonstrated anticancer activities against breast cancer through various mechanisms [15–23]. In addition, BD alone or combined with PectaSol-C® modified citrus pectin (MCP) inhibits growth and invasive behavior of the highly metastatic triple-negative human breast cancer cells in vitro and in vivo [24–26]. However, the effects of BD and its combination with TAM on ER-positive breast cancer have never been evaluated.

Based on the data described above, we investigated the sensitivity of ER-positive MCF-7 cells and its tumor xenografts to BD, 4-OHT/TAM and their combination treatment. Here we show, for the first time, that BD and 4-OHT/TAM work synergistically against breast cancer by suppressing estradiol-induced proliferation of MCF-7 cells in vitro and tumor growth in vivo, which related to induced apoptosis and regulation of TAM resistant proteins (p21/CDKN1A and Bcl-2) expression. The findings reveal a novel potential strategy against ER-positive human breast cancer using combination treatment of tamoxifen with BD.

## Methods

### Cell culture

A non-tumorigenic epithelial human breast cell line MCF-10A, estrogen receptor (ER) -positive MCF-7 and ER-negative MDA-231 human breast cancer cell lines were obtained from ATCC (Manassas, VA, USA). MCF-10A were cultured in DMEM/F12 containing 10% horse serum, epidermal growth factor (EGF, 20 ng/ml), hydrocortisone (0.5 mg/ml). cholera toxin (100 ng/ml), insulin (10 μg/ml) and penicillin (50 U/ml), streptomycin (50 U/ml). MCF-7 and MDA-231 cells were cultured in DMEM containing penicillin (50 U/ml), streptomycin (50 U/ml^−^) and 10% fetal bovine serum (FBS). For in vitro cell culture assays assessing the effect of BD on the ER activity, MCF-7 cells were stripped of steroids for 3 days before seeding by culturing in steroid-free medium (SFM): phenol red-free DMEM, supplemented with 10% newborn calf serum (NCS), penicillin (50 U ml^−1^), streptomycin (50 U ml^−1^) and 4 mM L-Glutamine. Medium, FBS, NCS and culture supplements were obtained from Gibco BRL (Grand Island, NY, USA).

### Chemicals and reagents

17β-estradiol (E_2_), 4-OHT, anastrozole, insulin, hydrocortisone, cholera toxin, and DMSO were purchased from Sigma (St. Louis, MO). EGF was purchased from Peprotech (Rocky Hill, NJ), horse serum was from Invitrogen (Carlsbad, CA). TAM pellets (5 mg/pellet, 60-day release) and E_2_ pellets (0.36 mg/pellet, 60-day release) were purchased from Innovative Research of America (Sarasota, FL, USA). Matrigel™ Matrix Growth Factor Reduced was purchased from BD Biosciences (Bedford, MA, USA). Anti-Raf-B, anti-p21, anti-Bcl-2, anti-Fibronectin and anti-β-actin antibodies were obtained from Santa Cruz Biotechnology (Santa Cruz, CA, USA). BreastDefend® (BD) was supplied by EcoNugenics, Inc. (Santa Rosa, CA, USA) and dissolved in DMSO at a concentration of 25 mg/ml then stored at −20 °C. The chemical composition of BD was previously published [26]. All other chemicals and reagents were of analytical grade.

### Cell proliferation assay

MCF-10A, MCF-7, MDA-231 cells were seeded into 96-well plates (5000 cell/well). After 24 h cells were treated with BD (10–50 μg/ml) for 3 days. Steroid-depleted MCF-7 cells were seeded into 96-well plates (5000 cell/well) in SFM. After 24 h, cells were treated with E_2_ (10 nM) plus 4-OHT (1 μM), BD (10–50 μg/ml) or a combination of both 4-OHT and BD for 3 and 6 days, respectively. Alternatively, MCF-7 cells were seeded into 96-well plates (5000 cell/well) in DMEM, and after 24 h treated with anastrozole (40 μM), BD (10 μg/ml) or a combination of both anastrozole and BD for 48 h, respectively. Cell proliferation was determined as described before [27]. Data points represent the mean ± SD in one representative experiment repeated at least twice.

### Determination of apoptosis

Steroid-depleted MCF-7 cells were seeded into 6-well plates (0.15 × 10^6^ cell/well) in SFM. After 24 h, monolayers were treated with E_2_ (10 nM) plus 4-OHT (1 μM), BD (10 μg/ml) or a combination of 4-OHT and BD for 6 days. Apoptosis induction was assessed by quantitation of cytoplasmic histone-associated DNA fragments using Cell Death Detection ELISA^PLUS^ Kit (Roche, Indianapolis, IN, USA). The manufacturer’s instructions were followed and data were expressed as the fold change *vs*. vehicle-treated cells (set equal to 1). Data points represent the mean ± SD in three independent experiments. Western blotting for PARP cleavage (c-PARP) was used to confirm the induction of apoptosis.

### Microarray gene expression profiling

Steroid-depleted MCF-7 cells were seeded into 6-well plates at a density of 0.15 × 10^6^ cell/well for 24 h and treated with E_2_ (10 nM) plus 4-OHT (1 μM), BD (10 μg/ml) or a combination of both for 6 days in SFM. Isolation, quantification, reverse transcription of RNA and TaqMan® Array Human Molecular Mechanisms of Cancer were performed as described before [28]. Relative quantity (RQ) of gene expression was normalized to *GAPDH* and determined using the 2^-∆∆Ct^ method [29]. Data were expressed as the fold change *vs*. vehicle-treated cells (set equal to 1) and represent the mean ± SD in three independent experiments.

### Western blot analysis

Steroid-depleted MCF-7 cells were seeded into 6-well plates (0.15 × 10^6^ cell/well) for 24 h and treated with E_2_ (10 nM) plus 4-OHT (1 μM), BD (10 μg/ml) or a combination of both for 6 days in SFM. Whole protein extracts isolated from cells were prepared and western blot analysis with anti-cleaved PARP, anti-Raf-B, anti-p21, anti-Bcl-2, anti-Fibronectin and anti-β-actin antibodies were performed as previously described [27]. Western blots were quantified with HP-Scanjet 550c and analyzed by UN-SCAN-IT software (Silk Scientific, Orem, UT, USA). Quantitative data composed of three independent experiments with statistical analysis were expressed as the fold change *vs*. vehicle-treated cells (set equal to 1) and added below or on the right of the representative blot images.

### Human breast tumor xenograft experiments

Nu/Nu immune-compromised female ovariectomized mice (4–5 weeks old) were obtained from Harlan Laboratories (Indianapolis, IN, USA) and maintained under specific pathogen-free conditions with phytoestrogen-free ad libitum food and water. After one week, MCF-7 cells (5 × 10^6^) suspension in 50 μl sterile PBS was mixed with 50 μl Matrigel™ and subcutaneously implanted into both sides of the inguinal mammary fat pad. E_2_ pellets (0.36 mg/pellet, 60-day release) were implanted using a 10-gauge trochar into right back between the ear and shoulder of all mice to permit tumors to form. Mice with palpable tumors (~60 mm^3^) were randomly assigned into four groups (*n* = 13): control, TAM, BD and TAM + BD (number of tumors 16–24 per group). TAM pellets (5 mg/pellet, 60-day release) were implanted subcutaneously into the left back between the ear and shoulder using a 10-gauge trochar. BD was suspended in water and administered by intragastrical gavage 5 times per week with 100 mg kg^−1^ of body weight for additional 4 weeks. During the treatment period, tumor sizes were measured 3 times per week with microcaliper and body weight was recorded at the same time. Tumor volumes were calculated with the standard formula: tumor volume (mm^3^) = L × W^2^ × 0.5, where L is the length and W is the width of the tumor. At the end of the experiment (Day 29), mice were euthanized by CO_2_ inhalation. Tumors were harvested, weighed and fixed in 10% neutral-buffered formalin at 4 °C for 24 h or snap frozen and stored separately in liquid nitrogen.

Animal experiments were conducted in accordance with the protocol approved by the Animal Research Committee at the Indiana University Health Methodist Hospital (protocol no. 2014–02).

### Apoptosis measurement in tumor xenograft

Formalin-fixed tumors were embedded in paraffin within 48 h and stained with hematoxylin and eosin (H&E). The slides were viewed using inverted microscope (Leica Microsystems, Wetzlar, Germany) and apoptosis in the viable tumor cell area was quantified by counting apoptotic bodies in four fields of view (20 ×) by two independent observers (*n* = 5-10).

### Immunohistochemistry

Paraffin-embedded tumor tissue sections were analyzed by immunohistochemistry using primary antibodies against B-raf or BRAF (Clone VE1, Spring Bioscience, Pleasanton, CA, USA), Bcl-2 (Clone 124, Dako, Carpinteria, CA, USA), p21 (C-19, Santa Cruz Biotechnology, Santa Cruz, CA, USA) and fibronectin (H-300, Santa Cruz Biotechnology, Santa Cruz, CA, USA). Sections were de-paraffinized, and rehydrated. Heat mediated (20 min at 100 °C, DAKO PT module) antigen retrieval was performed as follows for each antibody: DAKO high pH buffer for BRAF, Bcl-2 and fibronectin, while p21 was in DAKO low pH buffer. Endogenous peroxidase activity was blocked by H_2_O_2_ for 5 min and slides were then incubated with BRAF and Bcl-2 antibodies for 20 min; fibronectin and p21 for 30 min. In negative controls, the primary antibody was replaced with PBS. Secondary antibody (DAKO Flex system) for Bcl-2 and BRAF was added to the sections for an incubation time of 20 min; for p21 and fibronectin, incubated for 30 min with the Envision + R DAKO system. The stain was developed using diaminobenzidine (DAB) and the sections counterstained with hematoxylin.

Localization and intensity of immunoreactivities against all primary antibodies used were examined on slides by inverted microscope (Leica Microsystems, Wetzlar, Germany). For the immunohistochemical quantification, randomly selected images were analyzed in each animal per group (*n* = 10) by ImageJ [30].

### Statistical analysis

All the statistical analysis was carried out using SigmaPlot 11.2.0 (Systat Software Inc., San Jose, CA, USA). Data were presented as mean ± SD. Statistical comparisons between many groups of data were carried out by ANOVA with the all pairwise multiple comparison procedures (Holm-Sidak method) at overall significance level *p* < 0.05.

## Results

### Anti-proliferative and pro-apoptotic effects of 4-OHT are augmented by BD in ER-positive breast cancer cells MCF-7

**Fig. 1:**
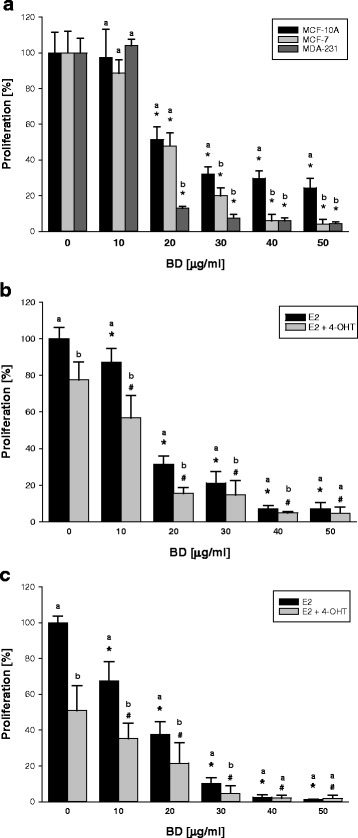
Effect of BD on the normal MCF-10A and breast cancer MCF-7 and MDA-231 cells. **a** MCF-10A, MCF-7 and MDA-231 cells were seeded and treated with BD (0–50 μg/ml) for 3 days. **b**, **c** MCF-7 cells were stripped of steroids for 3 days before seeding by culturing in steroid-free medium. After 24 h seeded into 96-well plates, cells were treated with E_2_ (10 nM) plus 4-OHT (1 μM), BD (0–50 μg/ml) or combination of 4-OHT and BD for **b** 3 days and **c** 6 days, respectively. Cell proliferation was determined by MTT assay. Each bar represents the mean ± SD of triplicate. Similar results were obtained in three independent experiments. Statistical analysis by ANOVA and Holm-Sidak. **a** * *P < 0.05* BD vs control (0 μg/ml) for different cell lines. **b**, **c** * *P < 0.05* BD vs control (0 μg/ml) in E_2_ group, # *P < 0.05* BD vs control (0 μg/ml) in E_2_ + 4-OHT group, different letters above bars indicate significant differences *P < 0.05* for the same concentration of BD

To evaluate the effect of BD on normal human epithelial mammary gland cells and ER-negative MCF-7 and ER-positive human breast cancer cells we treated MCF-10A, MCF-7 and MDA-231 cells with BD (10 – 50 μg/ml) for 3 days. Here we show that the low concentration of BD only slightly decreased proliferation of normal breast cells MCF-10A, whereas BD strongly suppressed growth of breast cancer cells MCF-7 and MDA-231 (Fig. 1a).

To evaluate the effect of BD on the proliferation of the estrogen-dependent ER-positive MCF-7 cells, we depleted these cells of estrogen and treated MCF-7 cells with E_2_, BD and 4-OHT as described in Materials and Methods. Our results show that after 3 and 6 days of treatment, 4-OHT (1 μM), ER receptor antagonist, significantly inhibits E_2_ -dependent proliferation of MCF-7. Moreover, BD further enhances anti-proliferative effect of BD in a dose- and time-dependent manner (Fig. 1b-c). Thus, 10 μg/ml of BD in the combination with 1 μM of 4-OHT was selected for future experiments in vitro. As shown in Fig. 1a, E_2_-independent proliferation of MCF-7 cells was not sensitive to the inhibitory effects BD at lower concentrations of BD.

**Fig. 2:**
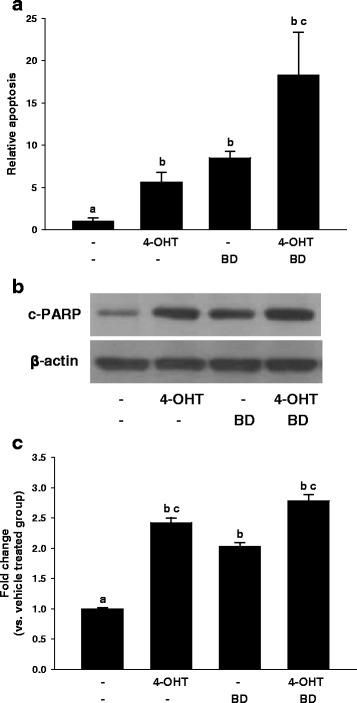
Effect of 4-OHT and BD on apoptosis of MCF-7 human breast cancer cells. MCF-7 cells were seeded and treated as described in Fig. 1c for 6 days. Apoptosis was evaluated by Cell Death Detection ELISA (**a**) and western blotting for the expression of c-PARP (**b**). Representative blots show expression of c-PARP and β-actin was used as loading control. Three independent experiments were done for the western blot studies and quantitative data with statistical analysis were shown below the representative blot image (**c**). Statistical analysis by ANOVA and Holm-Sidak. Different letters above bars indicate significant differences *P < 0.05*. The graphical data represent mean +/− SD

To determine whether the inhibition of cell proliferation by 4-OHT and BD are associated with apoptosis, we evaluated whether 4-OHT, BD and their combination induce nuclear DNA fragmentation [31]. As shown in Fig. 2a, there were nearly 18 fold increases in the apoptosis with 4-OHT (1 μM) and BD (10 μg/ml) combination compared to the vehicle-treated control after 6 days. Apoptosis induction in MCF-7 cells was further confirmed by western blotting for the cleaved fragment of PARP (c-PARP) [32, 33], where a combination of 4-OHT and BD distinctly increased the amount of c-PARP (Fig. 2b-c).

In addition, we treated MCF-7 cells with BD (10 μg/ml) and an aromatase inhibitor anastrozole (40 μM) or their combinations for 48 h. Although anastrozole inhibited proliferation of MCF-7 cells by 6% and BD by 13%, their combination suppressed proliferation by 39%, suggesting possible synergistic effect (Additional file 1: Figure S1). Since the major focus of the current study was on the evaluation of the combined effect of BD and tamoxifen, we will address the combined effect of BD and anastrozole in our future study.

### 4-OHT and BD combination regulates multiple genes related to apoptosis and TAM resistance

To further investigate molecular mechanisms underlying enhanced apoptosis induced by 4-OHT and BD combination, gene expression profiling with microarrays was carried out using total RNA from MCF-7 cells treated with 4-OHT and BD. Each set of four experiments was done in triplicate to increase the precision of estimation. The overlapping regulated genes with large recurring expression differences (at least 1.6-fold changed) compared to the vehicle-treated control after treatment of 6 days are summarized (Tab. 1). Based on the literature search, several genes associated to apoptosis and TAM resistance were identified, such as upregulation of genes encoding v-raf murine sarcoma viral oncogene homolog B (*BRAF*), caspase 9 (*CASP9*), and downregulation of genes encoding B-cell CLL/lymphoma 2 (*Bcl-2*), fibronectin 1 (*FN1*). Moreover, 4-OHT and BD combination showed maximal effects comparing with individual treatment, which might be the reason of enhanced apoptosis. Upon closer inspection, we found several genes that are regulated differently by BD versus 4-OHT. For example, *CDKN1A*, which encoding cyclin-dependent kinase inhibitor 1A (p21), is upregulated by both BD and combination, but not by 4-OHT.

**Table 1:** Combination of 4-OHT with BD regulates expression of cancer progression related genes

Gene	Description	4-OHT (RQ)	BD (RQ)	Combination (RQ)
*BRAF*	serine/threonine-protein kinase B-Raf	2.91 ± 0.55*	1.85 ± 0.10	5.27 ± 0.82*
*PTK2B*	protein tyrosine kinase 2 beta	2.76 ± 0.62	2.25 ± 1.33	4.51 ± 1.41*
*CDKN1A*	cyclin-dependent kinase inhibitor 1A (p21)	1.06 ± 0.14	3.26 ± 0.28*	4.06 ± 0.22*
*NFKBIA*	NF-kappa-B inhibitor	1.86 ± 0.50	2.35 ± 0.33*	3.42 ± 0.16*
*FYN*	FYN oncogene related to SRC, FGR, YES	1.81 ± 0.34	2.36 ± 0.81	3.16 ± 0.81*
*CASP9*	caspase 9	2.04 ± 0.33	1.67 ± 0.16	2.67 ± 0.67*
*BCL2*	B-cell CLL/lymphoma 2	0.13 ± 0.10*	0.29 ± 0.23*	0.08 ± 0.05*
*CCND2*	cyclin D2	0.39 ± 0.08*	0.12 ± 0.10*	0.10 ± 0.10*
*FN1*	fibronectin 1	0.41 ± 0.04*	0.30 ± 0.09*	0.19 ± 0.11*
*ITGA2B*	integrin, alpha 2b	0.62 ± 0.12*	0.58 ± 0.20*	0.41 ± 0.12*

DNA-microarray analysis was performed on TaqMan® Array Human Molecular Mechanisms of Cancer as described in *Materials and Methods*. MCF-7 cells were stripped of steroids for 3 days before seeding by culturing in steroid-free medium. After 24 h seeded into 6-well plates, cells were treated with E_2_ (10 nM) plus 4-OHT (1 μM), BD (10 μg/ml) or a combination of both for 6 days in steroid-free medium. Data are the means ± SD of three independent experiments. Analysis of the RQ gene expression data was performed using the 2^-∆∆CT^ method. Statistical analysis by ANOVA *P < 0.05

**Fig. 3:**
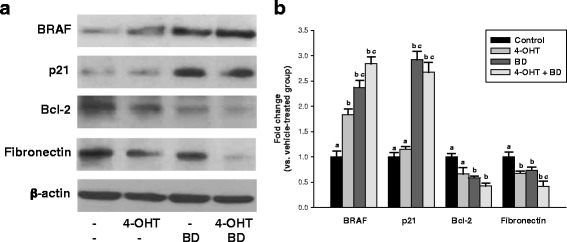
Combination of 4-OHT with BD regulates expression of apoptosis related proteins. **a** MCF-7 cells were treated for 6 days as described in Fig. 1. Whole protein extracts isolated from cells were prepared and western blot analysis with anti-Raf-B, anti-p21, anti-Bcl-2, anti-Fibronectin and anti-β-actin antibodies were performed as described in *Materials and Methods*. β-actin was used as loading control and representative blots from three experiments were shown. **b** Quantitative data composed of all the experiments in MCF-7 cells with statistical analysis were on the right of the representative blot image. Statistical analysis by ANOVA and Holm-Sidak. Different letters above bars indicate significant differences *P < 0.05*. The graphical data represent mean +/− SD

We evaluated if 4-OHT and BD combination affects expressions of genes, which involved in apoptosis and TAM resistance, at the translation level as well. Consistent with gene expression microarray data obtained at the mRNA level after treatment of 6 days, induction of BRAF, p21, and suppression of FN1, Bcl-2 in MCF-7 cells were detected by western blot analysis (Fig. 3a). Results of quantification indicated that expression of BRAF is induced nearly 2.4 fold with BD, 1.8 fold with 4-OHT and 2.8 fold with combination; expression of Bcl-2/FN1is suppressed around 0.6 fold/0.7 fold with BD, 0.7 fold with 4-OHT and 0.4 fold with combination; expression of p21 is induced nearly 2.9 fold with BD and 2.7 fold with combination (Fig. 3b).

### TAM and BD co-treatment inhibits growth of tumor xenografts by induction of apoptosis

We have recently demonstrated that BD is not toxic in vivo. An intragastric gavage of BD (100 mg/kg of body weight for 4 weeks) did not affect body weight or activity in liver enzymes and did not show any sign of toxicity in liver, spleen, kidney, lung and heart tissues in mice [26].

**Fig. 4:**
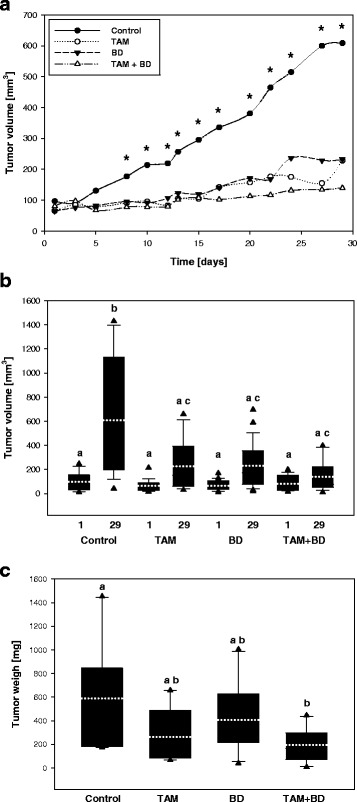
Inhibition of human breast tumor growth by TAM, BD, and TAM and BD combination *in vivo*. **a** Xenograft experiments were performed as described in *Materials and Methods*. During the treatment period, tumor sizes were measured 3 times per week. Statistical analysis by ANOVA and Holm-Sidak. *P < 0.05: control vs TAM, control vs. BD, control vs, BD + TAM. (*n* = 16-24 tumors per group). **b** Tumor sizes at the beginning (Day1) and the end (Day 29) of the treatment. Statistical analysis by ANOVA and Holm-Sidak. Different letters above bars indicate significant differences *P < 0.05*. Box plots represent 5th/10th percentiles, mean (*white dotted line*), horizontal bars represent median values, whiskers indicate minimum to maximum values and triangles represent outliers. **c** At the end of the experiment (Day 29), tumors were harvested and weighed. Statistical analysis by ANOVA and Holm-Sidak. Different letters above bars indicate significant differences *P < 0.05*. Box plots represent 5th/10th percentiles, mean (*white dotted line*), horizontal bars represent median values, whiskers indicate minimum to maximum values and triangles represent outliers

To determine the effect of TAM and BD in vivo, the growth of ER-positive human breast tumor xenografts was monitored in ovariectomized nude mice subcutaneously injected with MCF-7 cells and treated with TAM, BD or the combination of TAM and BD, as described in Materials and Methods. The control group (E_2_ alone) exhibited rapid growth of MCF-7 tumors from day 7 and treatments of BD or TAM resulted in the significant inhibition of tumor growth when compared to control. In addition, the combination of TAM and BD further enhanced inhibitory effect of TAM on tumor growth (Fig. 4a). On day 29, the average tumor volume ± SD in the combination group (TAM and BD) was approximately 139 ± 121 mm^3^, which was nearly 77% of inhibition compared with control (~608 ± 489 mm^3^) (Fig. 4a). Although the treatment of TAM or BD also significantly suppressed tumor volume (TAM ~ 228 ± 216 mm^3^, BD ~ 232 ± 180 mm^3^), their combination suppressed only slightly tumor volume on day 29 (TAM and BD ~139 ± 121 mm^3^) (Fig. 4b). In addition, there were no significant differences in body weight between control and treatment groups (data not shown). Interestingly, mice in the combination group were more relax, active and healthy than mice in the TAM group (data not shown). In addition, average tumor weight in the combination group (TAM and BD) (195 ± 141 mg) was decreased by 67% when compared to the control group (587 ± 469 mg) at the end of treatment period on day 29 (Fig. 4c).

**Fig. 5:**
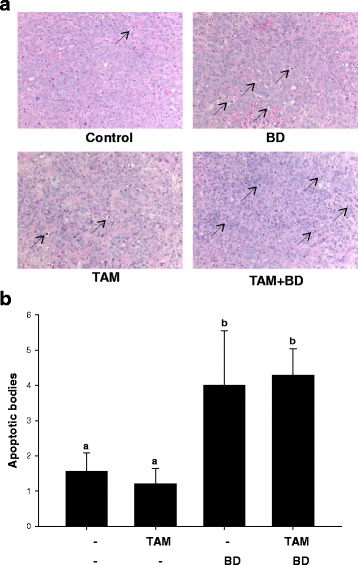
Induction of apoptosis in human breast tumor xenografts. **a** Representative H&E staining of apoptotic bodies in MCF-7 human breast tumors, **b** quantification was determined as described in *Materials and Methods*. Statistical analysis by ANOVA and Holm-Sidak. Different letters above bars indicate significant differences *P < 0.05*. The graphical data represent mean +/− SD. (*n* = 5–10)

To determine if TAM and BD combination inhibits growth of ER-positive human breast tumor by enhancing apoptosis in vivo, we quantified the amounts of apoptotic bodies in tumor xenografts. As seen in Fig. 5a, more apoptotic bodies were detected in the tumor tissues from TAM and TAM and BD combination treated groups compared with the control group. Although there was no statistical difference between TAM and control group, TAM and BD combination showed maximal increase of apoptotic bodies (64%) compared with control, indicating that suppression of tumor growth can be attributed to the induction of apoptosis in cancer cells (Fig. 2). Although the number of apoptotic bodies in breast tumors is suggestive for the induction of apoptosis it is necessary to confirm apoptosis by other method, as is the expression of specific pro-apoptotic protein Bcl-2 in tumors.

### Effect of BD and TAM on the apoptotic and TAM resistant proteins expression in tumors

**Fig. 6:**
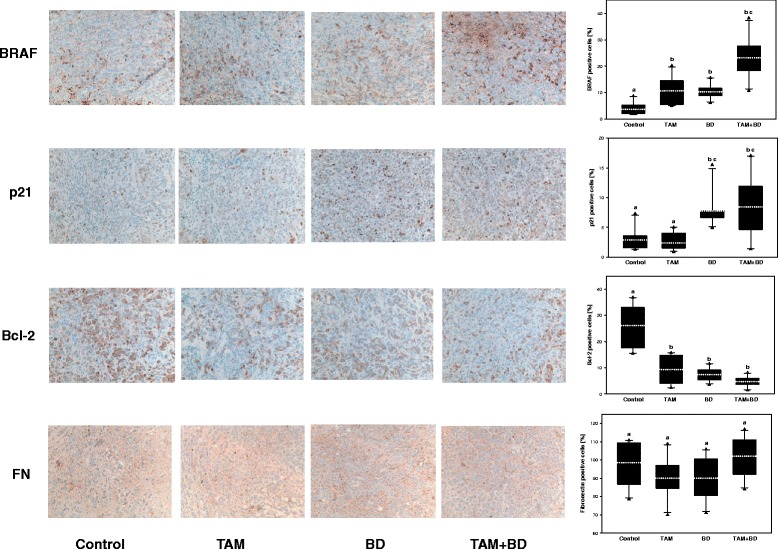
Combination of TAM with BD regulates expression of apoptosis and TAM resistance related proteins in human breast tumors. Animal experiments were performed as described in Fig. 3. Paraffin-embedded tumor tissue sections were analyzed by immunohistochemistry using antibodies against BRAF, p21, Bcl-2 and Fibronectin (FN). Representative localization and intensity of immunoreactivities against all primary antibodies are shown. Immunohistochemical quantification of BRAF, p21, Bcl-2 and FN were determined as described in *Materials and Methods*. Statistical analysis by ANOVA and Holm-Sidak. Different letters above bars indicate significant differences *P < 0.05*. The graphical data represent mean (*white dotted line*) +/− SD, triangles represent outliers. (*n* = 10)

To assess whether the mediated tumor growth inhibition is associated with the expression of proteins involved in apoptosis and TAM resistance, tumor tissues from TAM, BD and TAM and BD combination treated mice were subjected to immunohistochemistry. As shown in Fig. 6, TAM and BD combination markedly induced expressions of BRAF and p21, whereas expression of pro- apoptotic Bcl-2 protein was decreased compared to the vehicle-treated control. Similar results were found in western blot analysis of tumor tissues as well (data not shown). These in vivo observations are in accordance with our in vitro data, with MCF-7 cells treated with BD, 4-OHT or the combination of BD and 4-OHT. However, the expression of Fibronectin was not affected in the breast cancer tumors.

## Discussion

TAM has been a frontline treatment for both early and advanced ER-positive breast cancer in pre- and post-menopausal women [34–36]. A new therapeutic strategy is focus on the combination with other agents that increase efficacy and decrease toxicity of TAM. Here, we evaluate the therapeutic potential of co-treatment of TAM with BD, a natural dietary supplement, in ER-positive human breast cancer. Our results indicate that the combination of 4-OHT and BD or the combination of TAM and BD resulted in the suppression of cell and tumor growth and induction of apoptosis in vitro and in vivo, respectively. Microarray, western blot and immunohistochemistry analyses further demonstrate that the combination treatment regulates expression of proteins involved in the cancer growth and cell death. Importantly, TAM and BD co-treatment significantly suppresses tumor growth in vivo.

The emergence of TAM resistance is almost inevitable, which pose a major clinical problem. Mechanisms may include changes in the expression or function of ER, variation in ER-associated transcription factor recruitment, altered expression of specific microRNAs, and genetic polymorphisms involved in TAM metabolic activity [37, 38]. Among of them, ER plays the major role in driving resistance [39]. It has been shown that the enhanced cell proliferation and reduced susceptibility to cell death mediated by ER signaling are in part through the regulation of p21, a key cell cycle break, and Bcl-2, the major anti-apoptotic and pro-survival protein [40, 41]. Recently, Raha et al. established *de novo* and acquired TAM-resistant breast cancer models, which exhibit reduced p21 and elevated Bcl-2 expression [37]. In clinical studies, loss of p21 is associated with a TAM growth-inducing phenotype and increased Bcl-2 expression is an important phenomenon in metastatic TAM-resistant breast tumors [42, 43]. Our data demonstrated that 4-OHT/TAM alone had no effect on the expression of p21, but BD and/or combination with 4-OHT/TAM resulted in significant upregulation of p21. Moreover, addition of BD to 4-OHT/TAM leads to enhanced inhibition of Bcl-2. Altered expression of these key proteins may attribute to quercetin, a bioflavonoid presented in BD, which inhibits proliferation and induces apoptosis in ER-positive breast cancer cells via upregulation of p21 and downregulation of Bcl-2 protein expression [44, 45]. In addition, Oh et al. demonstrate that quercetin suppresses angiogenesis in TAM-resistant breast cancer through inhibition of Pin1 [46]. Therefore, BD may reverse TAM resistance by enhanced inhibition of Bcl-2 and significant induction of p21, which driving cells into apoptosis. Although we found positive effects in the inhibition of proliferation and induction of apoptosis which was associated with the altered gene expression in MCF-7 cells treated with BD and 4-OH/TAM, these effects were determined in only one ER-positive human breast cancer cells and xenograft model. Therefore, it is possible to expect that other ER-positive human breast cancer cells would also respond to this treatment. Nevertheless, since each cancer cell type has specific and unique genetic make-up, it is plausible that other set of genes would be associated with the anticancer activity of BD and 4-OH/TAM. We have previously demonstrated that therapeutic activity of BD itself was associated with the expression of genes associated with proliferation and metastasis in highly invasive human breast cancer cells MDA-MB-231 and in an animal model of breast-to-lung cancer metastasis [24, 26]. Another crucial aspect in gene targeting is a temporal gene expression. In our current study, we analyzed gene expression at 6 days because at this time point we detected significant response of BD and 4-OH/TAM in the inhibition of proliferation and induction of apoptosis in MCF-7 cells. Although it is important to evaluate also other time points, in vivo data confirmed the original cell culture data, increased expression of BRAF and p21 and decreased levels of Bcl-2 in tumors after the combined treatment in mouse after 29 days. Indeed, a temporal gene analysis and the use of other human breast cancer cells is necessary for the evaluation of specific molecular targets of BD and their combinations with typical breast cancer drugs. However, these analyses are behind the scope of the present manuscript and will be performed in future studies.

Hormonal therapy using TAM results in menopausal symptoms and serious symptoms not only greatly decrease the quality of life, but also may lead to discontinuation of the treatment [47, 48]. Hence, non-prescription dietary supplements are often used to relieve TAM-induced side effects. 3,3′-diindolylmethane (DIM), another purified components in BD, is the major product of indole-3-carbinol (I3C) in vivo and has promising activities against ER-positive breast cancer [49]. Katchamart et al. demonstrated a significant reduction in the *N*-oxygenation of TAM catalyzed by liver microsomes in rats fed with DIM, which may actually decrease the toxicity of TAM. Based on the marked shift in the metabolic profiles of TAM, they hypothesize that patients taking TAM in concert with administration of DIM dietary supplements could modulate the risk of developing toxic side effects if there is a similar alteration in humans [50]. *Ganoderma lucidum*, a medicinal mushroom in BD, has been used in Asian countries to improve health and promote longevity for centuries [15]. A pilot clinical trial suggests that spore powder of *G. lucidum* may have beneficial effects on cancer-related fatigue and quality of life in ER-positive breast cancer patients undergoing endocrine therapy [51]. In our present study, mice in the combination group exhibit less fatigue and more energy comparing mice in the TAM group. Definitely, more rigorous experiments are needed to confirm the findings and clarify the specific mechanisms behind them.

## Conclusions

Our study is the first report describing the combination effects of TAM and BD in ER-positive human breast cancer. BD sensitizes breast cancer cells to 4-OHT/TAM treatment in vitro and in vivo, promotes apoptosis, interferes with multiple pathways important for TAM resistance and has potential in decrease TAM-induced side effects. Therefore, BD could decrease future TAM resistance in the combination therapy in the originally anti-estrogen responsive breast cancers. Thus, BD may be recommended as novel adjuvant polybotanical preparation for patients with ER-positive breast cancer undergoing conventional endocrine therapy. More ER-positive breast cancer cell models will be employed in the further study and clinical trials exploring efficiency of BD are required to support its use in breast cancer patients.

## Additional file

Additional file 1: Figure S1. Effect of BD and anastrozole on MCF-7 breast cancer cells. (PPTX 39 kb)

## Abbreviations

4-OHT: 4-hydroxytamoxifen
BD: BreastDefend
ER: Estrogen receptor
TAM: Tamoxifen
