# Supplementary material for: BreastDefend enhances effect of tamoxifen in estrogen receptor-positive human breast cancer in vitro and in vivo
Source: BMC Complement Altern Med. 2017 Feb 16;17:115. doi: 10.1186/s12906-017-1621-7 (PMC5314617; doi:10.1186/s12906-017-1621-7)
Supplement: Additional file 1: Figure S1. — Effect of BD and anastrozole on MCF-7 breast cancer cells. (PPTX 39 kb) [file 12906_2017_1621_MOESM1_ESM.pptx]

## Slide 1
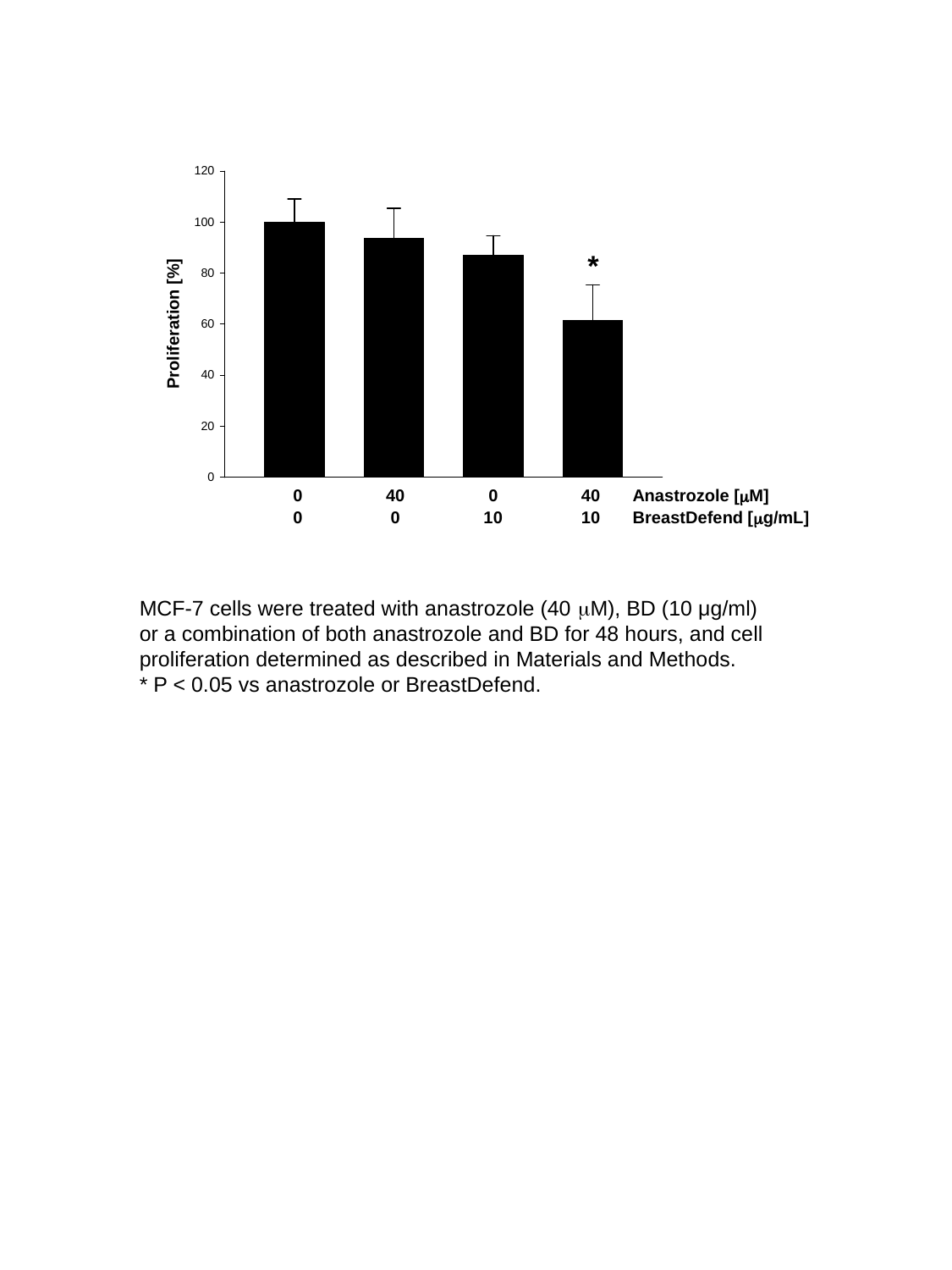

MCF-7 cells were treated with anastrozole (40 mM), BD (10 μg/ml)
or a combination of both anastrozole and BD for 48 hours, and cell proliferation determined as described in Materials and Methods.
* P < 0.05 vs anastrozole or BreastDefend.
